# Data on gene cloning, expression, purification, and characterization of the glycoside hydrolase family 11 from *Bacillus velezensis*

**DOI:** 10.1016/j.dib.2023.109834

**Published:** 2023-11-19

**Authors:** Dinh Minh Tran, To Uyen Huynh, Thi Huyen Nguyen, Tu Oanh Do, Anh Dzung Nguyen

**Affiliations:** Institute of Biotechnology and Environment, Tay Nguyen University, Buon Ma Thuot, Dak Lak 630000, Viet Nam

**Keywords:** Dataset, *Bacillus velezensis* RB.IBE29, Xylanase gene, Recombinant family 11 xylanase

## Abstract

*Bacillus velezensis* RB.IBE29 is a chitinolytic bacterium originally isolated from the rhizospheric soil of black pepper grown in Vietnam. This bacterium is a strong biocontrol agent against plant pathogens and possesses a novel chitinase system. Genome sequences available in CAZy database revealed *B. velezensis* possesses one gene encoding xylanase belonging to glycoside hydrolase family 11; however, this enzyme has yet to be un-experimentally characterized. In this work, *xyA* gene was isolated from the genomic DNA of strain RB.IBE29 and cloned in *Escherichia coli* DH5α cells using the pUC19 vector. Sequencing analysis showed that the ORF of *xyA* contains 642 bp and encodes the deduced xylanase with 213 aa and 23.27 kDa. The domain structure of the enzyme has a signal peptide and a family 11 catalytic domain. *xyA* (without peptide sequence) was successfully expressed in *E. coli* BL21-CodonPlus (DE3)-RIPL cells using the pColdII vector and purified using the HisTrap FF column. Purified recombinant xylanase degraded xylan substrates, had the highest hydrolytic activity at 55°C in 20 mM sodium phosphate buffer (pH 6.0), and MgCl_2_, CoCl_2_, and MnCl_2_ enhanced the enzymatic activity. Nucleotide sequence of xyA was submitted to the DDBJ/GenBank/EMBL under accession number LC779040. This is the first data on the gene cloning, expression, purification, and characterization of the glycoside hydrolase family 11 from *B. velezensis*.

Specifications Table

**Table:** 

Subject	Microbiology: Applied Microbiology
Specific subject area	Molecular biology, Biotechnology
Data format	Raw, Analyzed
Type of data	Figures
Data collection	- Isolation of *xyA* gene from *B. velezensis* RB.IBE29 - Cloning of *xyA* in *Escherichia coli* DH5α cells using the pUC19 cloning vector and sequencing of the insert - Analysis of nucleotide sequence of *xyA* and primary structure of xylanase - Expression of *xyA* in *E. coli* BL21-CodonPlus (DE3)-RIPL cells using the pColdII expression vector - Purification of recombinant xylanase using the HisTrap FF column - Characterization of purified recombinant xylanase
Data source location	• Institution: Institute of Biotechnology and Environment, Tay Nguyen University • City/Province/Region: Buon Ma Thuot/Dak Lak/The Central Highlands • Country: Vietnam
Data accessibility	1. Nucleotide sequence Repository name: DDBJ/GenBank/EMBL Data identification number: Accession number LC779040 Direct URL to data: https://www.ncbi.nlm.nih.gov/nuccore/LC779040 2. Protein sequence Repository name: DDBJ/GenBank/EMBL Data identification number: Accession number BES79751 Direct URL to data: https://www.ncbi.nlm.nih.gov/protein/2581250181 3. Other data are available with this publication

## 1 Value of the Data

• Data provide a protocol for gene cloning, expression, and purification of the glycoside hydrolase family 11 of *B. velezensis* RB.IBE29 in *E. coli* cells.
• Data provide basic bio-properties of the glycoside hydrolase family 11 of *B. velezensis* RB.IBE29.
• Data can be useful for comparing biological properties of the glycoside hydrolase family 11 of *B. velezensis* RB.IBE29 originating from the Central Highlands and others.
• Data can be useful for comparing biological properties of the glycoside hydrolase family 11 of *B. velezensis* and other bacterial species.

## 2 Background

*B. velezensis* RB.IBE29 is a chitinolytic bacterium isolated from the rhizospheric soil of black pepper in Vietnam. According to experimental findings, strain RB.IBE29 had a high chitin-degrading activity and reduced the growth of *Phytophthora*, a fungal pathogen of black pepper [1]. According to field tests, strain RB.IBE29 was a suitable choice for producing black pepper sustainably [2]. Additionally, strain RB.IBE29 possessed a novel chitinase system with two family 18 chitinases and an AA10 protein. These enzymes have been expressed in *Escherichia coli* cells. Purified recombinant chitinases strongly affected the germination of fungal spores and the hatching of nematode eggs [3], [4]. Genome sequences available in the CAZy database showed that *B. velezensis* possesses one gene encoding xylanase belonging to glycoside hydrolase family 11; however, this enzyme has been un-experimentally characterized. This work aimed to establish data on the gene cloning, expression, purification, and characterization of the glycoside hydrolase family 11 from *B. velezensis* RB.IBE29.

## 3 Data Description

**Fig. 1:**
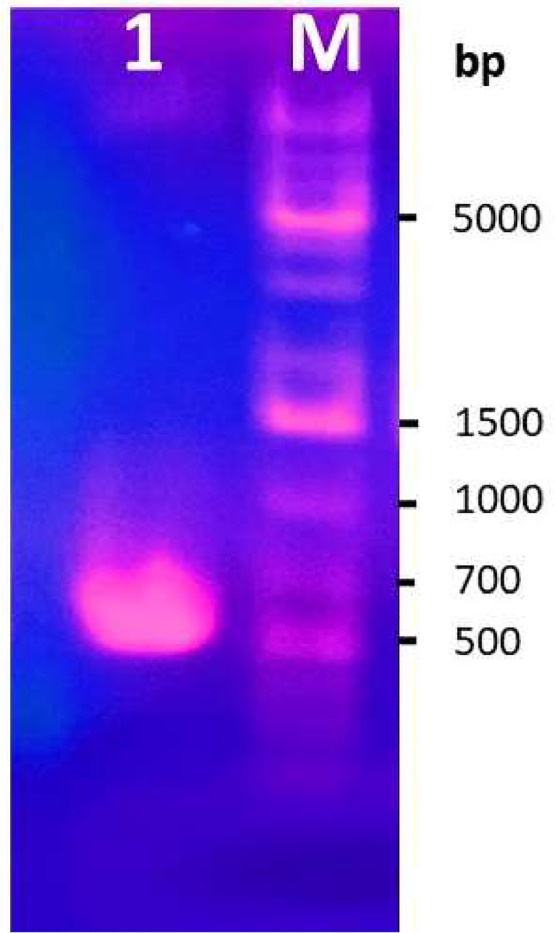
Isolation of *xyA* gene from *Bacillus velezensis* RB.IBE29. *Note:* Lane M, the DNA marker in bp; lane 1, the PCR product.

As shown in Fig. 1, the *xyA* encoding xylanase was identified and successfully isolated from the genomic DNA of the strain RB.IBE29 by PCR.

**Fig. 2:**
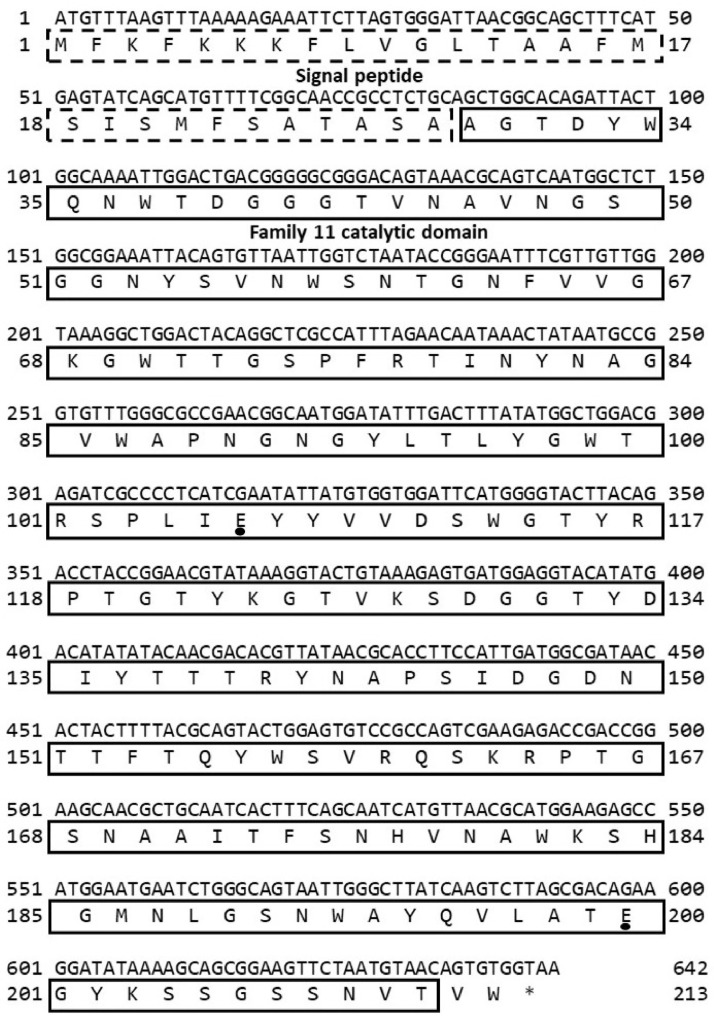
Nucleotide sequence of *xyA* gene and primary structure of xylanase of *Bacillus velezensis* RB.IBE29. *Note:* An asterisk indicates the stop codon, and closed circles indicate two catalytic residues.

**Fig. 3:**
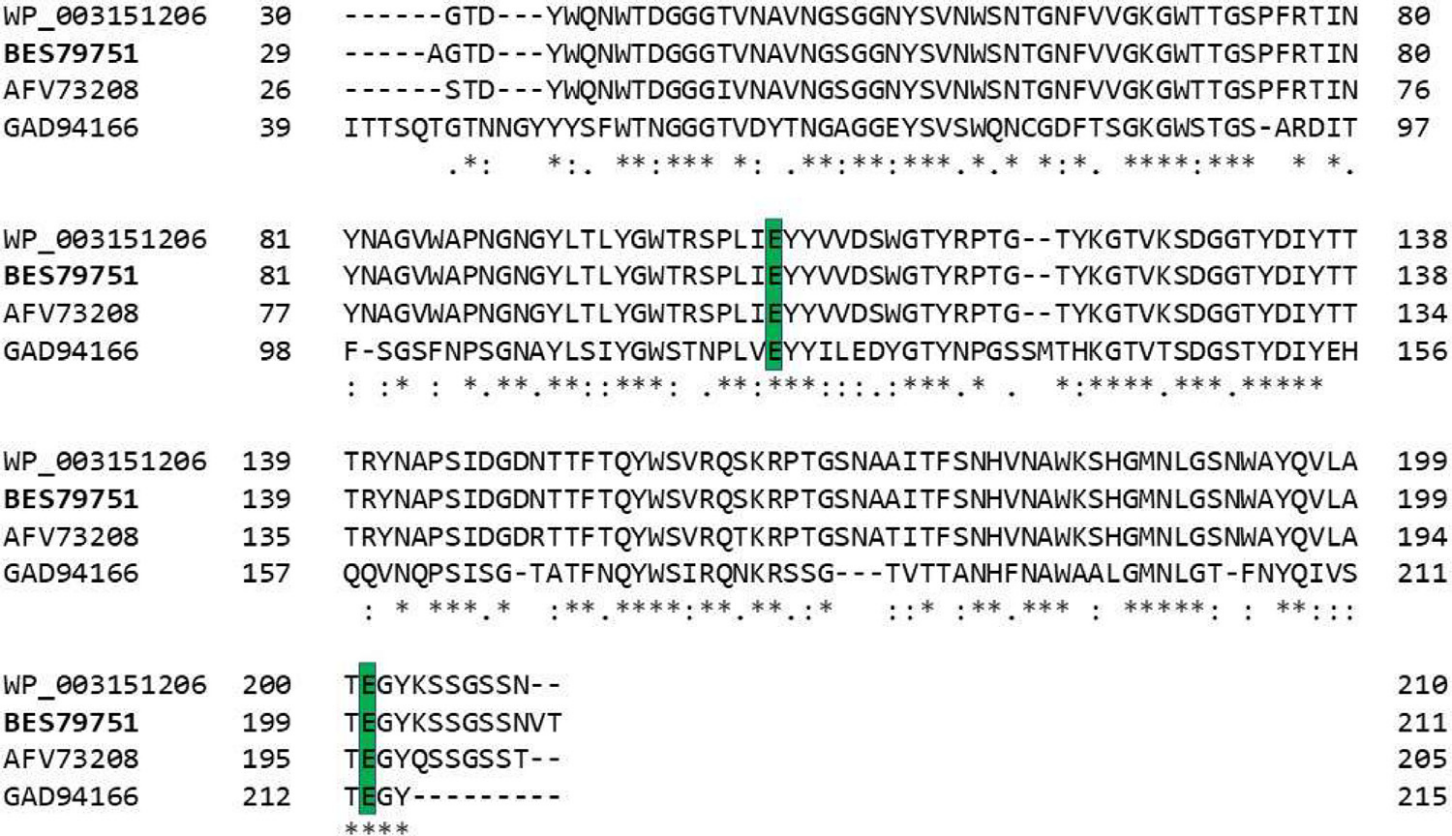
Alignment of amino acids in the catalytic domain of xylanase of *Bacillus velezensis* RB.IBE29 and those of other bacteria. *Note:* Asterisks indicate conserved amino acids; Green boxes indicate the two catalytic residues; WP_003151206, xylanase (WP_003151206) of *B. subtilis* 168; GAD94166, glycosyl hydrolases family 11 protein (GAD94166) of *P. variotii* No. 5; AFV73208, xylanase (AFV73208) of *B. subtilis* ABGx; and BES79751, xylanase (BES79751) of *Bacillus velezensis* RB.IBE29 (This work).

The purified PCR product was inserted into the pUC19 vector previously digested with SmaI, following which the nucleotide sequence of the gene was sequenced and analyzed. The ORF of *xyA* contains 642 base pairs (bp) and encodes a deduced protein of 213 amino acids (aa). The primary structure of the deduced protein was analyzed by using the SMART program. The result showed that the deduced protein contains a signal peptide sequence (28 aa, residues 1–28) at the N-terminus and a catalytic domain of family 11 xylanase (183 aa, residues 29–211) at the C-terminus (Fig. 2). The molecular size of the predicted enzyme is 23.27 kDa with signal peptide and 22.23 kDa without the signal peptide, respectively. The catalytic domain of deduced xylanase showed 100 % identity to that of characterized endo-1,4-beta-xylanase XynA (WP_003151206) of *B. subtilis* 168 [5], 100 % to uncharacterized glycosyl hydrolases family 11 protein (GAD94166) of *Paecilomyces variotii* No. 5 [6], and 96.15 % to xylanase (AFV73208) of *Bacillus subtilis* ABGx [7]. An alignment analysis showed that amino acids in the catalytic domain of the *B. velezensis* RB.IBE29 xylanase have a high sequence similarity to those of xylanases from other bacteria (Fig. 3). In addition, two glutamic acids, which were reported to be catalytic residues of family 11 xylanase [8], were found in the xylanase of strain RB.IBE29 (corresponding to Glu-106 and Glu-200) (Fig. 2 and 3). Nucleotide and protein sequences of the xylanase in this work were submitted to the DDBJ/GenBank/EMBL under accessions LC779040 and BES79751 and can be downloaded at https://www.ncbi.nlm.nih.gov/nuccore/LC779040 and https://www.ncbi.nlm.nih.gov/protein/2581250181, respectively.

**Fig. 4:**
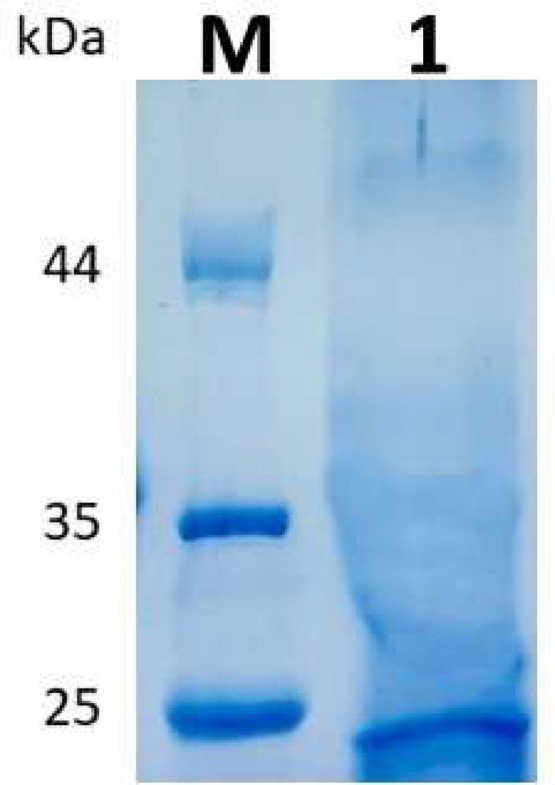
Purified recombinant xylanase of *Bacillus velezensis* RB.IBE29. *Note:* Five μg of purified recombinant xylanase was applied for SDS–PAGE analysis using an 11.5 % polyacrylamide gel. Lane M, size markers in kDa; lane 1, purified recombinant xylanase.

As shown in Fig. 4, *xyA* was successfully expressed in *E. coli* BL21-CodonPlus (DE3)-RIPL cells, and the resulting recombinant xylanase was purified. The molecular size of purified recombinant xylanase was estimated to be 24.5 kDa by SDS–PAGE. This estimation was very close to the protein's molecular size based on its amino acid sequence (xylanase without signal peptide, 22.23 kDa + polyhistidine tag, 3.18 kDa = 25.38 kDa).

**Fig. 5:**
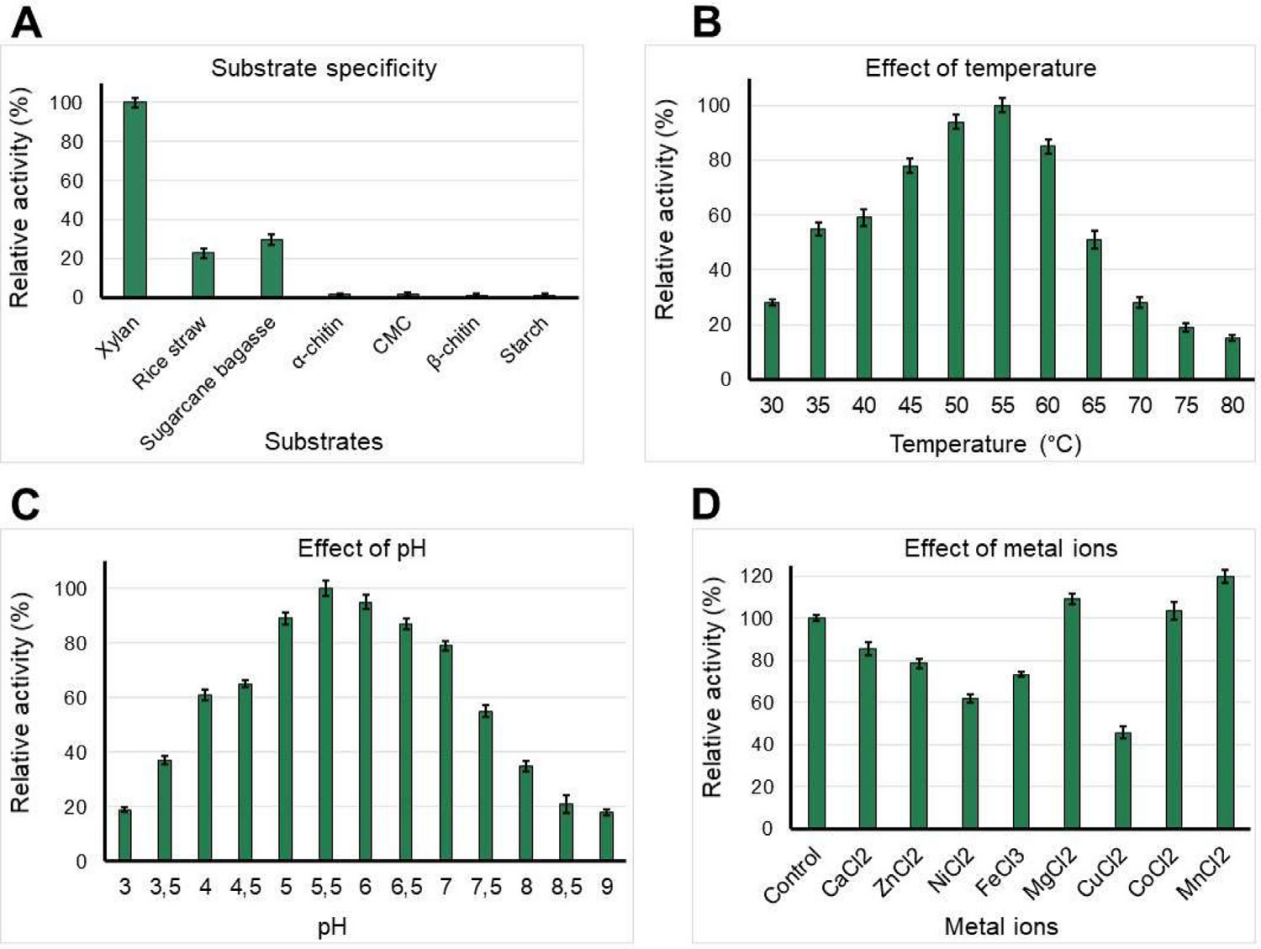
Bio-properties of purified recombinant xylanase of *Bacillus velezensis* RB.IBE29. *Note:* All results were the mean of triplicates and standard deviations.

Fig. 5A shows that the purified recombinant xylanase showed the highest substrate specificity toward xylan with 100 % relative activity (112.51 µmol reducing sugars), and very low activity toward CMC, chitin, and starch, respectively. As shown in Fig. 5B, temperatures ranging from 45 to 60 °C were suitable for xylanase activity; among them, 55 °C was the optimum temperature with 100 % relative activity (119.75 µmol reducing sugars). pH values ranging from 5.0 to 7.0 were suitable conditions for the hydrolysis of xylan by the purified xylanase, and pH 6.0 was shown to be the optimal pH of xylanase activity with 100 % relative activity (125.46 µmol reducing sugars) (Fig. 5C). Among the tested metal ions in this work, MgCl_2_, CoCl_2_, and MnCl_2_ enhanced enzymatic activity of the purified xylanase by 109.12, 103.56, and 119.75 % relative activity (135.6, 128.69, and 148.81 µmol reducing sugars), respectively (Fig. 5D).

## 4 Experimental Design, Materials and Methods

### 4.1 Bacterial strains, plasmids, and culture medium

*B. velezensis* RB.IBE29 [1] was used as a source for isolating the xylanase gene. *E. coli* DH5α (New England Biolabs, USA) and *E. coli* BL21-CodonPlus (DE3)-RIPL (Aligent, USA) were used as hosts. pUC19 (Thermo Fisher Scientific, USA) was used as a cloning vector, and pColdII (Takara, Japan) was used as an expression vector, respectively. Luria-Bertani (LB) medium was used for routine cultures.

### 4.2 Gene cloning and sequencing analysis

Gene cloning and analysis were conducted as described by Tran et al. [3], [4]. Briefly, the open reading frame of the *xyA*, which encodes the family 11 xylanase, was isolated from the genomic DNA of *B. velezensis* RB.IBE29 by PCR using forward primer 5’-ATGTTTAAGTTTAAAAAGAAATTCTTAG-3’, reverse primer 5’-TTACCACACTGTTACATTAGAACTTC-3’, and Phusion High-Fidelity DNA polymerase (Thermo Fisher Scientific, USA) per the supplier's instructions. Electrophoresis was used to separate the amplified products on a 1.2 % agarose gel (w/v). Using the Wizard SV Gel and PCR Clean-Up System (Promega Co., USA), the target band was purified. The vector pUC19 was incubated with the FastDigest SmaI (Thermo Fisher Scientific, USA), and the purified fragment was inserted into the SmaI site of the treated vector using the Mighty Mix (Takara, Japan) to create the recombinant vector pUC19-xyA. The recombinant vector pUC19-*xyA* was introduced into *E. coli* DH5α by heat shock. Transformants were cultured at 37 °C on LB agar plates containing X-Gal (0.04 mg/mL), ampicillin (100 μg/mL), and IPTG (Isopropyl *β*-D-thiogalactopyranoside, 0.1 mM). The recombinant vector pUC19-*xyA* from the positive colonies was examined by colony PCR, isolated, and purified using the GeneJET Plasmid Miniprep Kit (Thermo Fisher Scientific, USA). Inserts from purified recombinant vectors were sequenced at the 1^st^ BASE Company (Selangor, Malaysia). The signal peptide sequence of the deduced enzyme was predicted using SignalP 5.0 [9]. The domain structure was analyzed using the SMART database [10]. Molecular weight was calculated using the Compute pI/Mw tool [11]. The BLASTp program [12] was used to analyze the homology of the predicted enzyme and reported bacterial xylanases.

### 4.3 Expression and purification of recombinant xylanase

Expression and purification of recombinant xylanase were performed by Tran et al. [3], [4]. Briefly, the ORF (without signal peptide sequence, starting from Ala-29) of *xyA* was amplified by PCR using primers (forward primer 5’-GGTGGTGGATCCGCTGGCACAGATTACTG-3’ and reverse primer 5’-GGTGGTAAGCTTCCACACTGTTACATTAGAACTTCCGCTG-3’, underlines indicate cleavage sites of BamHI and HindIII, respectively), the pUC19-*xyA*, and the Phusion High-Fidelity DNA polymerase. After analyzing the amplified product by electrophoresis on a 1.2 % agarose gel (w/v), the target band was recovered and purified using the Wizard SV Gel and PCR Clean-Up System. After incubating the purified fragment with the FastDigest BamHI-HindIII (Thermo Fisher Scientific, USA), the Mighty Mix was used to ligate the treated insert into the BamHI-HindIII site of the vector pColdII, creating the recombinant plasmid pCold-xyA. The plasmid pCold-*xyA* was introduced into *E. coli* BL21-CodonPlus (DE3)-RIPL cells by heat shock. Transformants were grown at 33 °C on LB agar plates containing ampicillin (100 μg/mL). Recombinant vectors from the positive colonies were examined by colony PCR and then verified by sequencing. Cells from a positive colony were grown in 1.0 L of LB containing ampicillin at 33 °C with shaking until an OD600 nm of 0.6 was reached. The cells were induced by addition of IPTG to a final concentration 0.5 mM, and the culture was further incubated for an additional 33 h at 15 °C. Cultured cells were collected by centrifugation, resuspended in 125 mL of a buffer (20 mM sodium phosphate buffer (pH 7.4), 40 mM imidazole, and 0.5 M NaCl), and then sonicated using a Q700 sonicator (Qsonica, USA). The remaining cell debris was discarded by centrifugation, and a 1 mL HisTrap FF column (Cytiva, Sweden) was used to purify the recombinant xylanase present in the supernatant. Finally, SDS-PAGE was used to examine the eluted proteins.

### 4.4 Substrate Specificity of Purified Recombinant Protein

Xylanase activity was examined using various substrates, including xylan from beech wood, carboxymethyl cellulose (CMC), rice straw, sugarcane bagasse, α-chitin, β-chitin, and starch. A reaction mixture (900 µL) contained one of the substrates (1 % each, w/v) and 5 µg purified xylanase in 20 mM phosphate buffer (pH 6.0). In control, inactivated xylanase by incubating purified enzyme at 100 °C for 10 min was applied. The reaction mixture was incubated at 50 °C for 30 min, and then stopped with 500 µL of 3,5-dinitrosalicylic acid reagent. The reaction mixture was boiled at 100 °C for 5 min and reducing sugars generated from the reaction were measured at 540 nm with D-xylose used as the standard [13], [14], [15].

### 4.5 Effect of temperature on hydrolytic activity of purified recombinant xylanase

To examine the optimal temperature, the reaction mixture containing 1 % xylan from beech wood (w/v), 5 µg purified recombinant xylanase in 20 mM phosphate buffer (pH 6.0) was incubated at temperatures ranging from 30 to 80° C for 30 min [13], [14], [15].

### 4.6 Effect of pH on Hydrolytic Activity of Purified Recombinant Xylanase

To examine the optimal pH, the reaction mixture containing 1 % xylan from beech wood (w/v), 5 µg purified recombinant xylanase in various pH values was incubated at 55 °C for 30 min. Buffer systems were used in this test, including 20 mM glycine-HCl (pH 3.0–3.5), 20 mM sodium acetate buffer (pH 4.0–5.5), 20 mM phosphate buffer (pH 6.0–8.5), and 20 mM glycine-NaOH (pH 9.0) [13], [14], [15].

### 4.7 Effect of Metal Ions on Hydrolytic Activity of Purified Recombinant Xylanase

Effect of various metal ions on the activity of purified xylanase was measured by incubating purified recombinant xylanase (5 µg) with 5 mM of each metal ion (CaCl_2_, ZnCl_2_, NiCl_2_, FeCl_3_, MgCl_2_, CuCl_2_, CoCl_2_, and MnCl_2_) in 20 mM phosphate buffer (pH 6.0) containing 1 % xylan from beech wood (w/v) at 55 °C for 30 min [13], [14], [15].

## Limitations

Not applicable.

## Ethics Statement

The current work does not involve human subjects, animal experiments, or any data collected from social media platforms.

## CRediT authorship contribution statement

**Dinh Minh Tran:** Conceptualization, Methodology, Investigation, Formal analysis, Software, Data curation, Validation, Visualization, Writing – review & editing. **To Uyen Huynh:** Investigation, Formal analysis. **Thi Huyen Nguyen:** Investigation, Formal analysis. **Tu Oanh Do:** Investigation, Formal analysis. **Anh Dzung Nguyen:** Data curation, Validation, Visualization.

## Data Availability

Bacillus velezensis RB.IBE29 xyA gene for glycoside hydrolase family 11, complete cds (Original data) (NCBI) glycoside hydrolase family 11 [Bacillus velezensis] (Original data) (NCBI)
